# Understanding human genetic factors influencing primaquine safety and efficacy to guide primaquine roll-out in a pre-elimination setting in southern Africa

**DOI:** 10.1186/s12936-018-2271-z

**Published:** 2018-03-20

**Authors:** Shehu S. Awandu, Jaishree Raman, Takalani I. Makhanthisa, Philip Kruger, John Frean, Teun Bousema, Jandeli Niemand, Lyn-Marie Birkholtz

**Affiliations:** 1 Malaria Parasite Molecular Laboratory, Department of Biochemistry, Institute for Sustainable Malaria Control & MRC Collaborating Centre for Malaria Research, University of Pretoria, Private Bag x20, Hatfield, Pretoria, 0028 South Africa; 2 Centre for Emerging Zoonotic and Parasitic Diseases, National Institute for Communicable Diseases, Johannesburg, South Africa; 3 Wits Research Institute for Malaria Research, Faculty of Health Sciences, University of Witwatersrand, Johannesburg, South Africa; 4 Limpopo Malaria Control Programme, South African Department of Health, Limpopo, South Africa; 5 Department of Medical Microbiology, Radboud University Medical Centre, PO Box 9101, 6500 HB Nijmegen, The Netherlands

**Keywords:** Malaria, Primaquine, Malaria elimination, G6PD deficiency, CYP2D6

## Abstract

**Background:**

Primaquine (PQ) is recommended as an addition to standard malaria treatments in pre-elimination settings due to its pronounced activity against mature *Plasmodium falciparum* gametocytes, the parasite stage responsible for onward transmission to mosquitoes. However, PQ may trigger haemolysis in glucose-6-phosphate dehydrogenase (G6PD)-deficient individuals. Additional human genetic factors, including polymorphisms in the human cytochrome P450 2D6 (CYP2D6) complex, may negatively influence the efficacy of PQ. This study assessed the prevalence of G6PD deficiency and two important CYP2D6 variants in representative pre-elimination settings in South Africa, to inform malaria elimination strategies.

**Methods:**

Volunteers (n = 248) attending six primary health care facilities in a malaria-endemic region of South Africa were enrolled between October and November 2015. G6PD status was determined phenotypically, using a CareStart™ G6PD rapid diagnostic test (RDT), and genotypically for two common African G6PD variants, namely A+ (A376G) and A− (G202A, A542T, G680T & T968C) by PCR, restriction fragment length polymorphisms (RFLP) and DNA sequencing. CYP2D6*4 and CYP2D6*17 variants were determined with PCR and RFLP.

**Results:**

A prevalence of 13% (33/248) G6PD deficiency was observed in the cohort by G6PD RDT whilst by genotypic assessment, 32% (79/248) were A+ and 3.2% were A−, respectively. Among the male participants, 11% (6/55) were G6PD A− hemizygous; among females 1% (2/193) were G6PD A− homozygous and 16% (32/193) G6PD A− heterozygous. The strength of agreement between phenotyping and genotyping result was fair (Cohens Kappa *κ* = 0.310). The negative predictive value for the G6PD RDT for detecting hemizygous, homozygous and heterozygous individuals was 0.88 (95% CI 0.85–0.91), compared to the more sensitive genotyping. The CYP2D6*4 allele frequencies for CYP2D6*4 (inferred poor metabolizer phenotype) and CYP2D6*17 (inferred intermediate metabolizer phenotype) were 3.2 and 19.5%, respectively.

**Conclusions:**

Phenotypic and genotypic analyses both detected low prevalence of G6PD deficiency and the CYP2D6*4 variants. These findings, combined with increasing data confirming safety of single low-dose PQ in individuals with African variants of G6PD deficiency, supports the deployment of single low-dose PQ as a gametocytocidal drug. PQ would pose minimal risks to the study populations and could be a useful elimination strategy in the study area.

## Background

South Africa is one of eight southern African countries that are signatories of the Elimination 8 (E8) initiative, a multi-country coordinated effort to achieve malaria elimination. The four frontline countries (South Africa, Botswana, Namibia and Swaziland) aim for elimination, in various timeframes by 2020 [1], thus setting the stage for subsequent elimination in the other E8 member states [2]. In an effort to accelerate the region towards malaria elimination, novel parasite and integrated vector tools are being explored.

For treatment of patients with *Plasmodium falciparum* malaria, the World Health Organization (WHO) advocates the use of a single low-dose (0.25 mg/kg) of primaquine (PQ) together with artemisinin-based combination therapy in malaria-eliminating countries [3]. PQ is effective against the transmissible gametocyte stage of *P. falciparum* parasites and may reduce onward transmission to mosquitoes if deployed at community level [4, 5]. It is also the only commercially-available drug active against hypnozoites in *Plasmodium vivax* and *Plasmodium ovale* infections [6]. However, country-specific efforts towards low-dose PQ deployment for *P. falciparum* transmission reduction are being impeded by the perceived safety issue surrounding PQ use in glucose-6-phosphate dehydrogenase (G6PD)-deficient individuals. When exposed to high doses of 8-aminoquinoline anti-malarial agents like PQ and tafenoquine, G6PD-deficient individuals risk suffering haemolysis [6, 7]. However, at the recommended low dose, the risk of haemolysis is thought to be substantially reduced and PQ is considered safe [3].

G6PD deficiency is an X-linked genetic disorder that displays great polymorphism with about 186 described mutations [8], resulting in the most common enzymopathy in the world [7]. G6PD deficiency is clinically observed mainly in hemizygous males or homozygous females, with heterozygous females presenting with a wide spectrum of aberrant enzyme activities due to mosaicism. Circumstantial evidence suggests that G6PD deficiency imparts some resistance against malaria as evidenced by the overlap in the geographical distribution of the deficiency with present and past malaria endemicity [9], although the exact nature of such potential protection is unknown. Whilst earlier studies reported G6PD deficiency protection against severe forms of malaria [10, 11], recent data from a large multi-centre case control study and a meta-analysis suggests protection from cerebral malaria and high parasitaemia and not severe malarial anaemia, mainly in heterozygous individuals in African settings [12, 13]. Although the complexity of this disorder presents a diagnostic challenge, certain G6PD variants, including the African and Mediterranean variants, have been used to probe the general G6PD deficiency status in populations [14]. G6PD variants are classified according to their residual enzyme activity into three types [15]. These range from the most severe presentations but rare type 1, that present with congenital nonspherocytic anaemia, while type 2 are usually asymptomatic in the steady state until exposed to exogenous triggers. These type 2 variants are the equivalent of class II and III of the WHO classification system [8] and predisposes individuals to substantial risk of haemolytic anaemia. Type 2 variants include the Mediterranean, A−, Mahidol, Canton, Vanua Lava and Seattle variants amongst others [16]. Type 3 variants manifest phenotypically as normal G6PD activity with no clinical issues.

In sub-Saharan Africa, three variants are most commonly described, namely the B wild-type variant associated typically with normal G6PD activity and the G6PD African variants, A+ and A− [16]. The African variants (“A”) are amongst the most common in the world with a wide enzyme activity range [7]. The A+ variant carries an A376G mutation in exon V which results in mild enzyme deficiencies. By contrast, the more common A− variant which carries an additional mutation in exon IV (G202A) is associated mild clinical presentations such as jaundice and dark urine when exposed to 8-aminoquinolines [16]. Several other A− mutations (A542T, G680T and T968C) have been detected in West African individuals [17, 18].

PQ efficacy is further modulated by the human cytochrome P450 2D6 (CYP2D6) enzyme [19], which metabolizes ~ 25% of all clinically prescribed drugs [20]. CYP2D6 is highly polymorphic with varied enzymatic activity as a result of allelic sequence variations and protein structural rearrangements. These genotypic and phenotypic variations affect drug metabolism, probably increasing the risk of treatment failure or potential toxicity [21]. To date, more than 113 allelic variants (and sub-variants) of the *CYP2D6* gene have been reported [22], which can be classified in four distinct phenotypic groups. Poor metabolizers (PMs) have two copies of defective alleles resulting in reduced expression of a particular CYP2D6 enzyme. Intermediate metabolizers (IMs) are heterozygous for a defective allele and a functional allele resulting in slightly reduced enzyme activity. Extensive metabolizers (EMs) carry two functional alleles and have normal enzyme activity, while ultra-rapid metabolizers (UMs) have more than two functional gene copies and elevated enzyme activity [23]. The PMs with the CYP2D6*4 polymorphism are associated with PQ treatment failure in *P. vivax* [24], whereas IMs harbouring CYP2D6*17 mutations have been linked with diminished enzyme activity in black Africans [25]. Whilst the impact of CYP2D6 metabolizer status on the efficacy of single low-dose PQ for gametocyte clearance is currently unknown, it is conceivable that the gametocytocidal effect of PQ would be reduced if lower plasma concentrations of the active PQ metabolite are achieved [26].

Prevalence of G6PD deficiency and CYP2D6 polymorphisms in a population are thus important considerations for public health interventions involving the deployment of 8-aminoquinoline drugs like PQ [27]. In sub-Saharan Africa, prevalence of G6PD deficiency is estimated to range between 5 and 37.5% [9], and CYP2D6 allele frequencies range from 1 to 33% [28]. South Africa has previously (1940s–1980s) reported fewer cases of G6PD deficiency (5.3%) [29, 30] and CYP2D6 (2.6*%*) [31] mutations compared to its neighbouring countries, most likely a consequence of genetic differences among the distinct ethnic groups [32, 33]. However, recent population movements and immigration [34] may have led to shifts in the frequency of the enzyme deficiency and CYP2D6 variants across the country. Due to a paucity of current data, regionally-relevant evidence of G6PD deficiency and CYP2D6 variant metrics are needed. These will inform implementation of single low-dose PQ policy in pre-elimination settings within South Africa.

This study aimed to determine the current prevalence of both G6PD deficiency and CYP2D6 variants in representative populations in a malaria-endemic region of South Africa. G6PD deficiency measurements were also compared to phenotypic screening with a potential point-of-care tool in mass screen and test scenarios. This is the first study employing genotyping to evaluate locally relevant G6PD deficiency and CYP2D6 variants in a pre-elimination setting of South Africa.

## Methods

### Ethics statement

Ethical approval for the study was obtained from the ethical review committees of the University of Pretoria (Ethics reference No. 406/2014) and the Limpopo Department of Health (Ref 4/2/2). Signed informed consent in the local language (Tshivenda) was obtained from all participants prior to sample collection. Parents or guardians of children less than 7 years of age gave written, informed consent, while children between the ages of 7 and 17 years gave assent before sampling. An adult witness was required to co-sign the informed consent document.

### Study site and population

This study was conducted in the Vhembe District, a 25,597 km^2^ area in Limpopo Province, a malaria-endemic region in the northernmost part of South Africa. Malaria transmission primarily occurs during the months of September to May, with limited transmission generally occurring over the winter months of June and July [35]. Vhembe District is responsible for more than 60% of all reported malaria cases in South Africa [36], with 1543 cases and 18 deaths noted in the region in the 2015/16 transmission season, when sampling occurred [37, 38]. The district is bordered by Mozambique to the south-east, Zimbabwe to the north and Botswana to north-west. The climate is typically subtropical with mild winters and wet, warm summers with average rainfall per annum of 820 mm [39]. The district experiences frequent droughts with areas that are predominantly semi-arid [40].

**Fig. 1:**
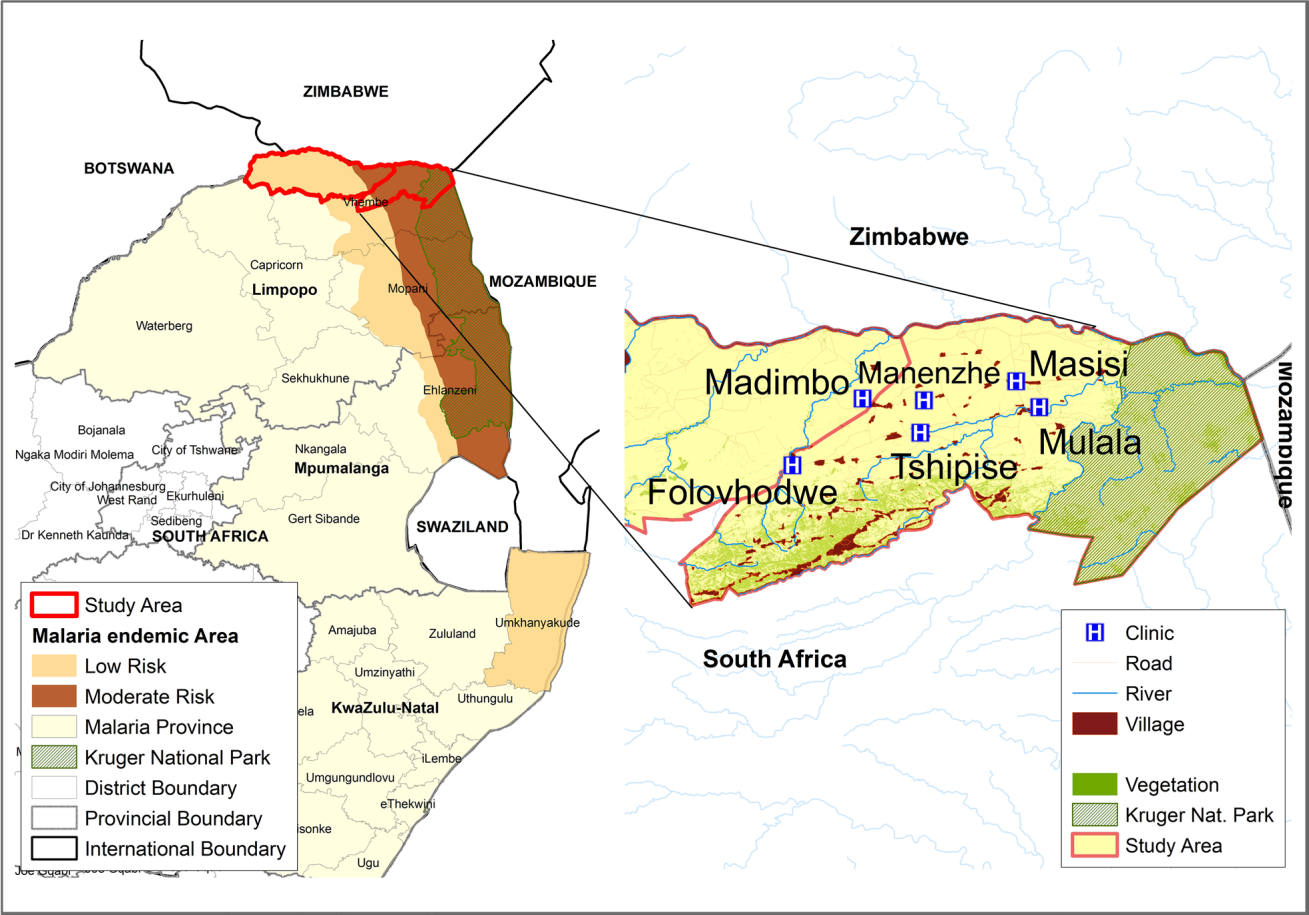
Map of the study site in Vhembe District, Limpopo Province, South Africa. The six primary health care clinics (H) and village catchment areas where participants were recruited are indicated. The study district in Limpopo Province is shown alongside other malaria-endemic provinces of KwaZulu-Natal and Mpumalanga, is in close proximity to Botswana, Zimbabwe and Mozambique

Study participants were recruited from six primary health care clinics (PHCs): Folovhodwe, Madimbo, Manenzhe, Masisi, Mulala and Tshipise in Mutale and Musina local municipalities (Fig. 1) during October and November 2015. Once recruited into the study in one particular clinic, patients were ineligible for subsequent enrolment even if they presented at a different clinic. The district has an estimated population of 1,347,235 with a population density of 52.6 persons per km^2^ [41]. The area is mainly inhabited by the Venda ethnic group, with sizable Zimbabwean immigrant populations (Shona and Ndebele ethnic groups) that work on the commercial farms and reside in the area.

### Questionnaire administration and sample collection

A brief questionnaire on demographics (specifically age and gender), travel history, history of fever or any malaria symptoms, ethnicity and residence was administered to the consenting participants. Upon completion of the questionnaire, finger prick filter paper (Munktell TFN card, Lasec, Cape Town, South Africa) blood spots were collected from the participants for *P. falciparum* parasite detection and human genotyping studies. Sample size in this pilot study was constrained by the availability of CareStart™ G6PDd rapid diagnostic test (RDT) kits.

### Malaria parasite detection

Peripheral finger prick blood samples were tested for *P. falciparum* parasite antigens using RDT kits detecting the histidine-rich protein 2 (First Response^®^ Malaria Antigen *P. falciparum* card test HRP2, Premier Medical Corporation, India) in accordance with the South African National Malaria Diagnosis Quality Assurance guidelines [42, 43]. The test was performed on individuals presenting with fever at the PHCs. The presence of *P. falciparum* parasites was confirmed by multiplex polymerase chain reaction (PCR) as previously described [44].

### G6PD phenotypic screening

The CareStart™ G6PD RDT (Access Bio Inc., New Jersey, USA) was administered concurrently with the malaria RDT on 248 participants at the PHCs as per the manufacturers’ guidelines. Briefly, a finger prick blood sample (2 µl) was collected through the sample pipette, applied into the sample well and 2 drops (~ 100 µl) of assay buffer added. The test was developed at room temperature (25 °C) for 10 min, after which the results were read by two blinded primary evaluators, with a third blind evaluator acting as a tiebreaker to make the final decision in case of discrepancies. Normal G6PD individuals were classified according to the development of a distinct purple coloured test band, while in G6PD-deficient patients, no purple coloured test band developed. A test was declared invalid when either no or incomplete blood migration took place.

### G6PD genotyping

DNA was extracted from blood-saturated filter paper discs of 3 mm diameter using the Chelex method [45]. Specific regions of the *G6PD* gene were amplified and subjected to restriction fragment length polymorphisms (RFLP, all restriction enzymes sourced from New England Biolabs, Inc., USA) to detect mutations as previously described [14]. A 308 base pair (bp) amplicon was amplified from genomic DNA and subjected to *Fok*I digestion for 60 min at 37 °C. Uncut fragments were classified as G6PD variant B, while samples where two fragments of 184 and 124 bp were produced, were classified as G6PD A+ variants. Only samples positive for G6PD A+ mutations underwent further PCR amplification and RFLP analysis to detect additional mutations. Subsequent digestion was performed with *N1a*III (G202A; 211, 81 and 130 bp), *Bsp*EI (G542T; 130, 80 and 50 bp), *Bst*NI (G680T; 115, 98 and 29 bp) and *Nci*I (T968C; 282, 162 and 120 bp) for 60 min at 37 °C (except for *Bst*NI where digestion occurred at 60 °C). All G6PD deficiency restriction products were resolved on 3% agarose/TAE gel with ethidium bromide staining. Images were captured on UVDoc HD2 transilluminator (UVITEC, Cambridge, UK).

### CYP2D6 genotyping

Genotyping of CYP2D6*4 was performed by PCR amplification of a 309 bp segment of the *CYP2D6* gene followed by overnight digestion with *Bst*NI at 60 °C as previously described [46], resulting in a 309 bp fragment for *wt/wt* CYP2D6*4; 309, 201 and 108 bp fragments for *wt/mt* CYP2D6*4 or 201 and 108 bp fragments for *mt/mt* CYP2D6*4. CYP2D6*17 genotyping was performed by PCR amplification of a segment of the *cyp2d6* gene of interest and subsequent overnight digestion with *Bts*CI as previously described [46], resulting in 407, 328, 79, 67 and 55 bp fragments for *wt/wt*. For CYP2D6*17 *wt/mt* the resulting fragment sizes were 407, 328, 79, 67 and 55 bp; *mt/mt* CYP2D6*17 resulted in 328, 79, 67 and 55 bp fragments. For all the CYP2D6 variants, the PCR and restriction products were resolved on 1.5% agarose/TAE with ethidium bromide staining gel. Images were captured on UVDoc HD2 transilluminator.

### Sanger sequencing

All G6PD A− positive PCR products were confirmed with Sanger nucleotide sequencing. After purification with the NucleoSpin^®^ Gel and PCR clean-up kit (Macherey–Nagel, Duren, Germany), samples resuspended in HiDi™ formamide were sequenced using an ABI PRISM BigDye^®^ terminator V3.1 cycle sequencing kit and processed on ABI PRISM 3130xl and ABI PRISM 3500xl genetic analyzers (Applied Biosystems™/Life Technologies, Carlsbad, USA). BLAST queries were conducted for preliminary identifications of the sequences and thereafter analysed with MAFFT v 7.182 and CLC sequence viewer software.

### Data analysis

Data were analysed with SPSS version 20 (IBM Corp. NY, USA). Descriptive statistics were used to report the demographics of the participants, with phenotype and genotype being presented per PHC. Differences in proportions of individuals carrying the G6PD deficiency, CYP2D6*4 and CYP2D6*17 wildtype and mutant alleles were compared by Chi squared (χ^2^) tests. Odds ratios (ORs) were used to assess any association of G6PD deficiency, CYP2D6*4 and CYP2D6*17 occurrence based on gender. To test for conformity with the Hardy–Weinberg equilibrium (HWE), allele frequencies were analysed both by PHC and overall. The expected female allele frequencies were calculated based on male genotype prevalence (A+, A− and B) then compared with observed values using Chi squared tests as described [14]. The negative predictive value (NPV) of the CareStart™ G6PD deficiency kit was determined using the A− variant PCR–RFLP genotyping results as the ‘gold standard’ [47]. Agreement between the two test results was evaluated using Cohen’s Kappa coefficient (*κ*), with the following scale used to score strength of agreement; 0.01−0.20 slight; 0.21–0.40 fair; 0.41–0.60 moderate; 0.61–0.80 substantial and 0.81–1.00 almost perfect [48]. A *P* value of < 0.05 was considered statistically significant.

## Results

### Study participant characteristics and malaria parasite detection

From the six PHCs, a total of 248 participants were recruited into the study. More female participants (n = 193, 78%) compared to male participants (n = 55, 22%) were enrolled, with study participants’ median ages of 31 years for females (range 1–94) and 21 years for males (range 1–81). The majority (97%, 241/248) of participants self-identified as South African Venda ethnicity. Other ethnic groups screened included Pedi (1.2%, 3/248) and Tsonga (0.4%, 1/248), with 1.2% (3/248) Shona from Zimbabwe. None of the participants harboured detectable *P. falciparum* parasites by either *P. falciparum* RDT or single-step, multiplex PCR.

### G6PD genotyping

**Fig. 2:**
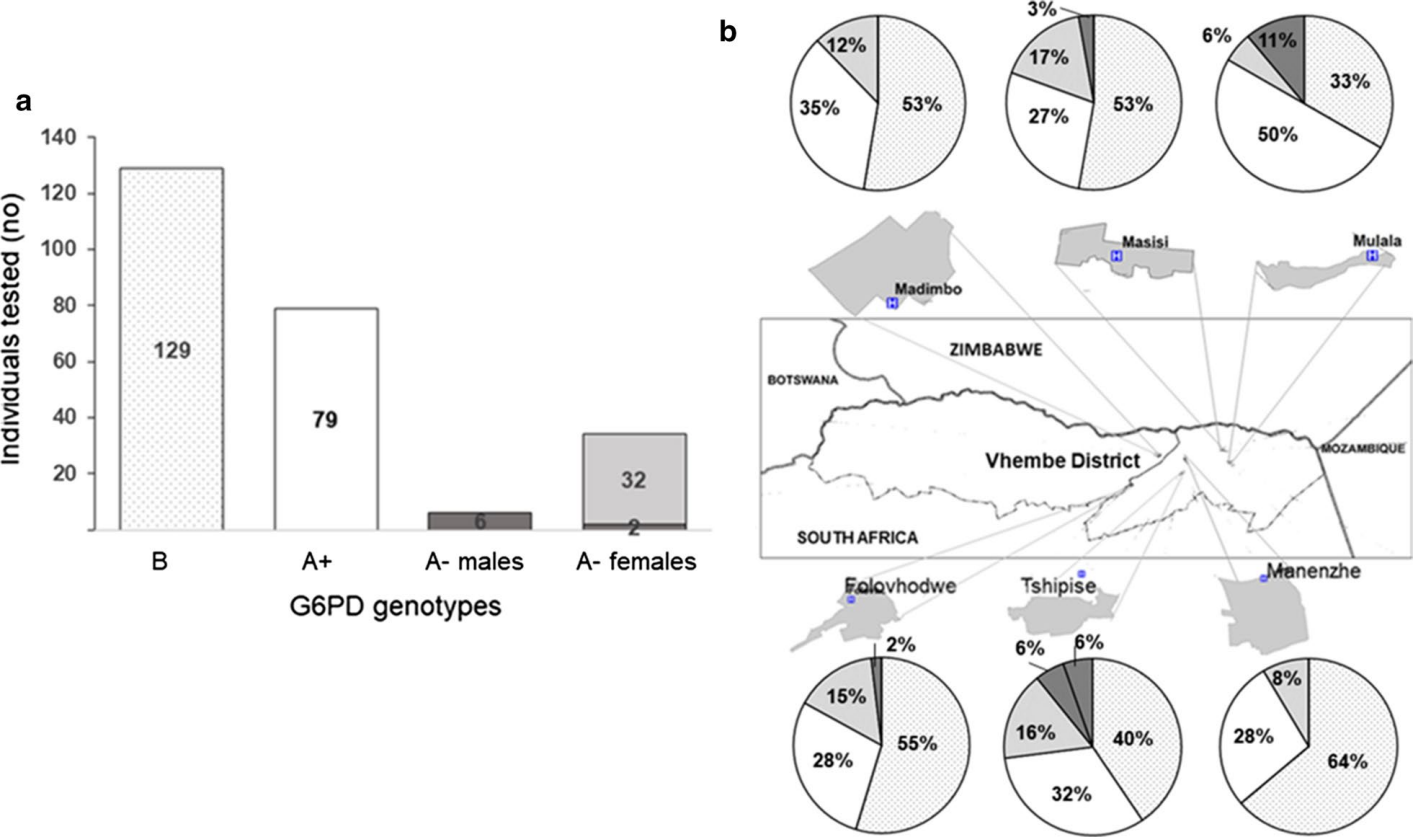
G6PD genotype prevalence. **a** G6PD genotypes (B patterned bar; A+ (A375G) white bar; A− (A375G/G202A) heterozygous light grey bar, hemizygous and homozygous dark grey bar) identified with PCR–RFLP and confirmed by sequencing for all the study participants. **b** G6PD deficiency stratified for each of the 6 primary health care clinics in Vhembe District, Limpopo Province, South Africa. (B patterned wedge; A+ (A375G) white wedge; A− (A375G/G202A) heterozygous light grey wedge, hemizygous and homozygous dark grey wedge)

The prevalence of the A+ and A− African G6PD variants among the study participants was determined by genotyping. Of the 248 participants screened, just over half (52%, 129/248) carried the G6PD normal B allele (Fig. 2a). The A376G mutation in exon V was detected in 32% (79/248) of the participants, identifying these individuals as carrying the A+ variant. A total of 13% (32/248) of the participants were G6PD A− heterozygous individuals. Only a minority of participants (3.2%, 8/248), were hemizygous or homozygous for the G6PD A− (A376G/G202A) variant. Among males, G6PD A− hemizygous individuals constituted 11% (6/55), while among females, 1% were G6PD A− homozygous (2/193). All G6PD A− variants identified contained A376G/G202A mutations, which was independently confirmed by DNA sequencing. The A542T, G680T or T968C mutations were not detected in the study population.

When comparing the proportions of participants with G6PD A+ and A− variants in the population, considerable variations were observed among localities (*P* = 0.01 for A+ and *P* = 0.05 for A− genotypes, respectively). In Manenzhe PHC, the highest proportion (64%) of G6PD B variants but lowest proportion (8%) of G6PD A− variants were detected. All the A− participants from Madimbo and Manenzhe PHC were A− heterozygous females (Fig. 2b). Mulala PHC recorded the highest proportions of A− hemizygous participants (11%) while homozygous participants (6%) were only identified from Tshipise PHC. All of the A− deficient participants G6PDd were of Venda ethnicity.

The genotypic frequencies were tested to confirm adherence to the HWE. According to the principle, the genetic variation in a population should remain constant from generation to generation in the absence of disturbing factors. The HWE measurement indicated that in general, female gene frequencies did not significantly differ from predicted male gene frequencies (χ^2^ > 0.05). However, departures from HWE were seen for the G6PD deficiency variants for the general population (χ^2^ = 0.007) and in Tshipise PHC (χ^2^ = 0.009) (Table 1) where all of the study homozygous participants were reported. When comparing G6PDd A + and G6PDd A- genotypes, no distinctive association with gender was observed *(P* = 0.107 and 0.441, respectively).

**Table 1:** Frequency of G6PD genotypes in 6 primary health care clinics in Vhembe District, Limpopo Province, South Africa

	Genotypes											HW *P*(χ^2^)
	Males				Females							
	No.	A^−^	A^+^	B	No.	A^−^A^−^	A^+^A^−^	BA^−^	A^+^A^+^	BB	BA^+^	
Folovhodwe	10	1	4	5	43	0	5	3	0	24	11	0.880
Madimbo	6	0	0	6	51	0	1	6	2	24	18	NC
Mananzhe	9	0	3	6	38	0	1	3	2	24	8	0.548
Masisi	11	1	2	8	25	0	1	5	1	11	7	0.197
Mulala	7	2	2	3	11	0	1	0	3	3	4	0.317
Tshipise	12	2	1	9	25	2	1	5	1	6	10	0.009*
Totals	55	6	12	37	193	2	10	22	9	92	58	0.007*

G6PD genotypes: male (X*Y) hemizygous deficient A−, male (XY) normal A+ or B; female (X*X*) homozygous deficient = A−/A−; female (X*X) heterozygous deficient = A+/A− or B/A−; and female (XX) normal = A+/A+, B/B or B/A+

NC = not calculated as all males tested in Madimbo clinic carried the G6PD B variant

* P values < 0.05 indicate significant differences from predicted G6PD allele frequencies in females based on male allele frequencies for A−, A+ and B alleles using the Hardy–Weinberg equation (χ^2^)

### Phenotypic G6PD screening

The CareStart™ G6PD deficiency RDT test kit was used for phenotypic evaluation of G6PD status in the test population, to explore its utility as point-of-care tool. The CareStart™ G6PD deficiency RDT identified 13% (33/248) of participants as G6PD-deficient (Table 2), with males showing a higher prevalence of G6PD deficiency (18%; 10/55) compared to females at 12% (23/193) (odds ratio 1.642; 95% confidence interval 0.7293–3.6992, *P* = 0.229). All the identified G6PD-deficient individuals bar one (Tsonga) were of Venda ethnic origin.

**Table 2:** G6PD deficiency status amongst males and females participants per primary health care clinic according to the CareStart™ G6PD RDT

CareStart™ G6PDd RDT classification^a^	Males (n, %)		Females (n, %)	
	Deficient (10, 18%)	Normal (45, 82%)	Deficient (23, 12%)^b^	Normal (170, 88%)^c^
Primary health care clinics^a^				
Folovhodwe	2 (4%)	8 (14%)	7 (4%)	36 (19%)
Madimbo	1 (2%)	5 (9%)	1 (0.5%)	50 (26%)
Manenzhe	1 (2%)	8 (14%)	5 (3%)	33 (17%)
Masisi	2 (4%)	9 (16%)	3 (1%)	22 (11%)
Mulala	2 (4%)	5 (9%)	5 (3%)	6 (3%)
Tshipise	2 (4%)	10 (18%)	2 (1%)	23 (12%)

^a^No of participants and (%) indicated

^b^From the deficient females by CareStart™ G6PD RDT test, 2 were homozygous, 7 heterozygous and 14 normal

^c^From the 170 normal females, 25 were heterozygous and 145 normal

The CareStart™ G6PD deficiency RDT was able to detect all the genetically-determined G6PD-deficient hemizygous males and homozygous females (Table 2) with the highest risk of haemolysis. Comparison of the RDT phenotyping results with that of the G6PD A− (A376G/G202A) PCR–RFLP genotyping indicated specificity of 91.3% (95% CI 86.7–94.8) with a fair Cohen’s Kappa agreement (*κ* = 0.310). A more substantial agreement was observed among the males (*κ* = 0.711) than among the females (*κ* = 0.202). The negative predictive value for identifying hemizygous, homozygous and heterozygous participants by RDT was 0.88 (95% CI 0.85–0.91) compared to genotyping.

### Cytochrome P450 genotype and phenotype distribution

Mutations in the *cyp2d6* gene that could interfere with PQ activation were evaluated on the 248 collected samples (Table 3). Allele frequencies of 0.03 and 0.19 were detected for the CYP2D6*4 and CYP2D6*17 alleles, respectively. Only 2 of the study participants (0.8%, 2/248) were classified as *mt/mt* PM with activity scores (AS) of 0 with a minor proportion (6%, 15/248) classified as *mt/mt* IM (AS of 0.5–1.0), using the AS metric as described [49]. The majority of the population (231/248, 93%) was classified as extensive or normal metabolizers. No association between gender and either the CYP2D*4 (χ^2^, *P* = 0.731) or CYP2D6*17 (χ^2^, *P* = 0.906) genotype was noted.

**Table 3:** Prevalence of CYP2D6*4 and CYP2D6*17 alleles and inferred phenotypes amongst study participants

CYP2D6 alleles	Enzyme activity	Heterozygote (*wt/mt*)	Homozygote (*mt/mt*)	Allele frequency	Activity score	Inferred phenotype
CYP2D6*4	Non-functional	12	2	0.0323	0	PM
CYP2D6*17	Reduced	67	15	0.1956	0.5–1	IM

The genotypes were determined by PCR–RFLP. Genotypes: wildtype/mutant (*wt/mt*) and mutant/mutant (*mt/mt*). All genotyping experiments were performed in duplicate in two independent experiments. Inferred phenotypes: intermediate metaboliser (IM) and poor metaboliser (PM)

## Discussion

This study reports on the prevalence of G6PD deficiency as well as CYP2D6*4 and CYP2D7*17 mutations in a southern African malaria pre-elimination setting, with the aim of informing a low-dose PQ policy. The G6PD A− prevalence of 11% among hemizygous males was comparable to that previously reported in South African Venda ethnic group [50]. Higher G6PD deficiency prevalence among males was previously reported in ethnic groups residing in close proximity to neighbouring Mozambique and Zimbabwe [30, 50], consistent with the observations in this study. These countries have higher malaria transmission intensity compared to South Africa, which could explain their higher G6PD deficiency prevalence of up to 19% in males [51, 52]. Modelling the median population estimates for G6PD allele frequency predict low frequencies of 3.3% for South Africa [15]. However, maps aggregate average frequency data to national levels and may mask sub-national variation and local heterogeneity in G6PD deficiency. By contrast, in Swaziland, a study on PQ pharmacovigilance in two health facilities reported the absence of G6PD deficiency amongst 102 participants [53].

The presence of A− (A376G/G202A) variant was confirmed in G6PD-deficient individuals, corroborating its previously-described widespread distribution [29, 50]. Other G6PD variants such as the A542T, G680T or T968C mutations previously reported in the Gambia and Senegal [17, 18], were not identified in the current southern African study population. This is consistent with findings from two large randomized controlled multicentre studies in Africa [14, 54]. While the study aimed to inclusively genotype different G6PD deficiency variants identified as important in African settings, the presence of additional G6PD deficiency variants in the locality cannot be ruled out. Future studies using more novel and robust genotyping methods are envisaged, as they could identify novel mutations that may impact PQ roll-out [32].

PQ is an important addition to standard malaria treatment regimen to aid in transmission reduction, especially for countries on the cusp of elimination [6]. PQ efficacy may be influenced by individual and ethnic genetic differences amongst populations [21, 55] and is reliant on metabolic activation. The PM and IM CYP2D6 phenotypes observed (3.2 and 19.5%, respectively) may result in PQ treatment failure at the recommended 0.25 mg/kg dose [56]. These findings on CYP2D6*4 allele frequency and predicted PM phenotype is consistent with earlier reported 1.3% [31] and 4% [57] prevalence among the Venda ethnic group. Indigenous African blacks have consistently been reported with a high frequency of the CYP2D6*17 allele, in agreement with reported data in this study [31, 33, 58].

As South Africa moves towards malaria elimination, novel interventions such as the WHO-recommended low-dose PQ strategy need further exploration. However, individuals with the CYP2D6*4 variants that may be unresponsive to PQ treatment and CYP2D*17 variants, who may require higher PQ dose to ensure therapeutic efficacy, is cause for concern in the country [56]. While there is little conclusive evidence on the effect of PQ on community–wide transmission if deployed in mass drug administration [59], efficacy studies point to marked reduction in duration of gametocyte carriage [4, 60]. The PQ-associated gametocyte clearance and its outcomes on mosquito infectiousness depends on malaria transmission settings and the choice of artemisinin combination therapy. It has been demonstrated that PQ has modest benefits on asymptomatic individuals with lower parasite densities prior to initiation of combination PQ treatment with either artemether-lumefantrine [26] or dihydoartemisinin-piperaquine [60]. That PQ should not be administered to infants aged < 6 months, breastfeeding women of infants aged < 6 months as well as pregnant women (South Africa has a 12% teenage pregnancy rate and higher fertility rates among non-urban women) [61], identifies the treatment gaps for PQ, with impact dependent on the proportion of the population treated [62].

Accumulating evidence from Africa [63–65] and Asia [66], suggests that single-dose PQ at 0.25 mg/kg as a gametocytocidal drug to block transmission would be safe in areas where G6PD deficiency A− and other type 2 variants predominates, comparable to the settings in this study site. Encouraging evidence from neighbouring Swaziland, a country with a similar G6PD deficiency prevalence profile to South Africa, suggests the roll-out of a low-dose PQ policy with no prior genetic profiling requirement, at least in none G6PD-deficient participants by RDT, is associated with good safety profiles [53]. However, stringent pharmacovigilance and patient monitoring systems should be put in place to mitigate any adverse events. Further, the low levels of CYP2D6 PM and IM is encouraging for the use of single low-dose PQ as an adjunct to current malaria control tools, and may accelerate efforts to eliminate malaria in South Africa.

This study was limited by the small sample size constrained by the availability of the G6PD RDT tests. Moreover, a potential selection bias towards females (78%) was present in the sample set, and findings may, therefore, not be directly extrapolated to estimate the overall population structure and deficiency prevalence in the study area, which can be rectified through more general population sampling, rather than sampling only at PHC facilities. Since the study only deployed a G6PD deficiency RDT, enzymatic activity validation is ultimately required to evaluated functional loss of G6PD activity to disentangle female heterozygotes from deficient and the non-deficient females. Female gender segregation would control for the profound effects of comparing male hemizygous and female heterozygous on diagnostic performance of G6PD tests [67].

## Conclusions

Phenotypic and genotypic analysis both detected a low prevalence of individuals carrying the G6PD A−, CYP2D6*4 PM variants and modest prevalence of the CYP2D6*17 IM variants in the study area. These preliminary findings suggest the deployment of single low-dose PQ poses a minimal risk to the area populations and could be useful in elimination strategies, in localized areas of South Africa.
